# *Cordyceps cicadae* Mycelia Ameliorate Cisplatin-Induced Acute Kidney Injury by Suppressing the TLR4/NF-*κ*B/MAPK and Activating the HO-1/Nrf2 and Sirt-1/AMPK Pathways in Mice

**DOI:** 10.1155/2020/7912763

**Published:** 2020-02-06

**Authors:** Jeng-Shyan Deng, Wen-Ping Jiang, Chin-Chu Chen, Li-Ya Lee, Pei-Ying Li, Wen-Chin Huang, Jung-Chun Liao, Hung-Yi Chen, Shyh-Shyun Huang, Guan-Jhong Huang

**Affiliations:** ^1^Department of Health and Nutrition Biotechnology, Asia University, Taichung, Taiwan; ^2^Department of Chinese Pharmaceutical Sciences and Chinese Medicine Resources, China Medical University, No. 91, Hsueh-Shih R., Taichung 40402, Taiwan; ^3^Grape King Biotechnology Inc., Taoyuan, Taiwan; ^4^Graduate Institute of Biomedical Sciences, School of Medicine, China Medical University, Taichung, Taiwan; ^5^School of Pharmacy, China Medical University, Taichung, Taiwan

## Abstract

Acute kidney injury (AKI) is a common clinical problem, characterized by a sudden loss of renal function, a high risk of death, and the eventual development of renal fibrosis and renal failure. *Cordyceps cicadae* is a traditional Chinese medicine with the potential function of kidney protection. We analyze two sputum extracts, a water extract (WCC), and an ethanol extract (ECC), to assess the potential of treating AKI in an animal model of kidney injury induced by cisplatin. A nephrotoxic mouse model was first established by intraperitoneal injection of cisplatin. Subsequently, WCC and ECC were orally administered in these mice. The results show that WCC and ECC significantly alleviated cisplatin-induced AKI renal histological changes, serum creatinine (CRE) and blood urea nitrogen (BUN) production, and the levels of NO, TNF-*α*, IL-1*β*, and IL-6. The levels of malondialdehyde (MDA) and glutathione (GSH) were suppressed by administration of WCC and ECC. However, WCC treatment prevented these changes significantly better than ECC treatment. In addition, Western blot data showed that WCC attenuated the cisplatin-induced protein expression of cyclooxygenase-2 (COX-2) and inducible NO synthase (iNOS), as well as inhibiting nuclear factor-kappa B (NF-*κ*B) and mitogen-activated protein kinase (MAPK) activation in the kidney tissues. Furthermore, WCC greatly inhibited the expression of Toll-like receptor 4 (TLR4) and cisplatin-induced NF-*κ*B activation, as well as dramatically increasing the production of antioxidative enzymes (i.e., superoxide dismutase (SOD), glutathione peroxidase (GPx), catalase, nuclear factor erythroid 2-related factor 2 (Nrf2), and heme oxygenase 1 (HO-1)), silent information regulator T1 (Sirt1), and p-AMP-activated protein kinase (AMPK) in the kidney tissues. In addition, we found that WCC increased the expression levels of the autophagy-related proteins LC3B and Beclin-1; proapoptotic proteins, including cleaved caspase-3 and cleaved poly (ADP-ribose) polymerase (PARP) 1; and organic anion transporters 1 (OAT1) and 3 (OAT3) in the kidney tissues. Finally, WCC, ECC, and two bioactive compounds—adenosine and N6-(2-hydroxyethyl) adenosine (HEA)—inhibited the production of nitrite oxide (NO) and intracellular reactive oxygen species (ROS) triggered by lipopolysaccharide- (LPS-) stimulated RAW264.7 macrophages *in vitro*. Collectively, WCC could provide a potential therapeutic candidate for the prevention of cisplatin-induced kidney injury through the inhibition of oxidative stress and inflammation.

## 1. Introduction

Acute kidney injury (AKI) is a disease with high morbidity and mortality, which is characterized by a rapid loss of kidney function [1]. The most common causes of AKI include nephrotoxic drugs, renal ischemia-reperfusion (IR), sepsis, cardiac disease, hypertension, and diabetes mellitus. It has been estimated that two million people worldwide die from AKI every year, and the risk of chronic kidney disease (CKD) has been shown to be greatly increased in patients who survive AKI [2]. However, no effective method or drug to prevent and cure AKI is available at present.

Cisplatin is a commonly used chemotherapy drug for the treatment of a variety of solid malignancies, including head and neck, breast, bladder, lung, ovarian, and testicular cancers [3]. However, the clinical application of cisplatin is limited by its serious side effects, such as nephrotoxicity, myelosuppression, allergic reactions, and peripheral neuropathy [4]. Although the molecular basis of cisplatin-induced renal toxicity in an animal model is not yet fully understood, current studies have suggested that it is associated with the mechanisms of oxidative stress, inflammation, hypoxia, vascular injury, and the activation of apoptotic pathways. It has been found that cisplatin induces a large number of free radicals in the kidney tissue, which damage cell structures and various cellular components, such as proteins, DNA, and cell membranes. Therefore, inhibition of lipid peroxidation and antioxidant defense is a hallmark of cisplatin-induced nephrotoxicity [5]. Reactive oxygen species (ROS) have been associated with cisplatin-induced nephrotoxicity, as ROS can induce massive production of the inflammatory cytokine tumor necrosis factor (TNF-*α*). Release of TNF-*α* induces other inflammatory cytokines and leads to inflammatory responses [6, 7]. These inflammatory mediators and oxidative stress can cause damage to renal tubular cells and kidney tissue. Therefore, inhibition of the inflammatory response and oxidative stress can provide a potential strategy to treat cisplatin-induced nephrotoxicity.

Oxidative stress signaling is important in enhancing inflammation through the activation of nuclear factor-*κ*B (NF-*κ*B) and mitogen-activated protein kinases (MAPKs) [8]. Cisplatin-induced AKI is closely related to the activity of lipid peroxidation and oxidative defense in the kidney. It has been found that the response of many cells to oxidative stress involves signaling proteins which act through the antioxidant response element (ARE) and the transcription factor erythrocyte 2-associated factor 2 (Nrf2) [9], which plays an important role in the regulation of the phase II gene. Nrf2-induced heme oxygenase-1 (HO-1) prevents cytotoxicity of various oxidative stress and inflammatory responses [10]. Therefore, activation of Nrf2 is considered to be an important molecular target for renal protection. However, the role of the Nrf2/HO-1 pathway remains unclear *in vivo*.

Adenosine 5′-monophosphate-activated protein kinase (AMPK) is a crucial kinase involved in maintaining cellular energy homeostasis. Silent information regulator T1 (Sirt1), a NAD^+^-dependent histone deacetylase, plays diverse roles in stress resistance, apoptosis, senescence, aging, and inflammation [11]. Evolving data have suggested that the AMPK/Sirt1 pathway plays an important role in renal inflammation and can act as a therapeutic target for inflammation-associated diseases [12].

Autophagy is a typical cell death pathway which has been related to many pathological conditions and can serve to resist stress and, consequently, promote cell survival [13]. In addition, autophagy can pass on metabolic substrates to enable cells to meet their energy requirements, thereby accelerating cell growth and survival [14]. Therefore, an autophagy activator may be an effective drug in preventing and treating cisplatin-induced inflammatory responses. Organic anion transporters play an important role in the distribution and excretion of drugs, especially the organic anion transporters 1 (OAT1) and 3 (OAT3), which are highly expressed in the kidney and play an important role in the elimination of a series of substrate molecules by the kidney [15].

*Cordyceps cicadae*, also known as the “cicada flower,” is a parasitic fungus belonging to the family Clavicipitaceae which grows mostly on the larvae of *Cicada flammata* Dist. It has similar medical properties and effects to *C. sinensis* [16]. *C. cicadae* has been documented in many Chinese medicine prescriptions for the treatment of palpitations, epilepsy, convulsions, and several eye diseases. Recently, *C. cicadae* has been shown to have a variety of pharmacological activities, including renal interstitial fibrosis, antihypoglycemia, antitumor, antianalgesic, anti-inflammatory, antifatigue, and immunoregulatory effects [16]. Adenosine and N6-(2-hydroxyethyl) adenosine (HEA) are the main active components of *C. cicadae*. The pharmaceutical activities of adenosine include anti-inflammatory, neuroprotective, antiangiogenic, hypolipidemic, anticonvulsant, antioxidant, and immunomodulatory effects [17]. HEA is a Ca^2+^ antagonist and an anti-inflammatory agent associated with control of the brain and coronary circulation [18]. Signal transduction pathways have been suggested to explain the activation of inflammatory mediators after cisplatin-induced kidney injury. Therefore, we hypothesized that the TLR4/NF-*κ*B/MAPK, Nrf2/HO-1, and AMPK/Sirt1 signaling pathways are involved in the inflammatory mechanisms of WCC after cisplatin-induced kidney injury. In the present study, we attempt to determine the anti-inflammatory and antioxidant effects of the extract of *C. cicadae* mycelia in a newly developed mouse model with cisplatin-induced kidney injury. The results suggest that *C. cicadae* mycelia display potential as a dietary supplement for preventing AKI and inhibiting oxidative stress and inflammation.

## 2. Material and Methods

### 2.1. Reagents

Cisplatin, amifostine (AMF), adenosine, and N6-(2′-hydroxyethyl) adenosine (HEA), as well as other reagents and solvents, were obtained from Sigma-Aldrich (St. Louis, MO, USA). Commercial assay kits for BUN and CRE were provided by HUMAN Diagnostics Worldwide (Wiesbaden, Germany). Mouse TNF-*α*, IL-1*β*, and IL-6 ELISA Max™ Set Deluxe Kits were purchased from BioLegend Inc. (San Diego, CA, USA). Primary antibodies for Western blots against anti-COX-2, anti-p-JNK, anti-catalase, anti-GPx, anti-SOD, anti-AMPK, anti-Sirt1, anti-TLR4, anti-P62, anti-Beclin-1, anti-cleaved PARP, and anti-cleaved caspase were purchased from GeneTex (San Antonio, TX, USA). Anti-OAT1 and anti-OAT3 were purchased from ABclonal (MA, USA). Antibodies against anti-JNK, anti-p-ERK, anti-ERK, anti-p-p38, anti-p-I*κ*B*α*, and anti-LC3B were purchased from Cell Signaling Technology (Beverly, MA, USA). Antibodies against anti-iNOS, anti-NF-*κ*B, anti-I*κ*B*α*, anti-p38, anti-HO-1, anti-Nrf-2, and *β*-actin were purchased from Abcam (Cambridge, UK). Protein assay kits (Bio-Rad Laboratories Ltd., Watford, Herts, UK) were obtained as indicated.

### 2.2. Fungus Material

The lyophilized powder of *C. cicadae* mycelium was supplied by the Biotechnology Center of Grape King Inc. (Taoyuan, Taiwan).

### 2.3. Extract Preparation

The mycelium fermentation broth was taken, concentrated under reduced pressure at 55°C, lyophilized, and ground. An appropriate amount of powder was separately added to water and ethanol for extraction. Water extracts of *C. cicadae* mycelia were extracted with 100°C water and filtered. The filtrate was collected and lyophilized to dryness. In addition, the mycelia were extracted twice with 95% ethanol over a 7-day period and dried by rotary evaporation.

### 2.4. Cell Viability

Cell viability was based on the measured cell metabolism using an MTT assay. Cells (2 × 10^5^) were plated in a 96-well plate (three replicates) and allowed to attach overnight to become almost confluent. The cells were treated with samples in the presence of 100 ng/mL of LPS for 24 or 1 h. Then, MTT (0.5 mg/mL) was added to each well for an additional 4 h in media. The absorbance was measured at 570 nm. Survival percentage was calculated as percentage compared to control.

### 2.5. Nitrite Assay

Determination of nitrite concentration was performed by a colorimetric method based on the Griess reaction [19]. Briefly, the Griess reagent was added to 100 *μ*L supernatant of the cultured media, and the solutions were mixed and incubated for 10 min at 540 nm with an ELISA reader (Molecular Devices, Orleans Drive, Sunnyvale, CA).

### 2.6. Animals

Male ICR mice (6–8 weeks old, weight 20–25 g) were supplied by BioLASCO Taiwan Co., Ltd. (Taipei, Taiwan). The animals were maintained under a 12 h light/dark cycle with food and water ad libitum, at a relative humidity of 60 ± 10%, and at a temperature of 23 ± 2°C for 1 week before the experiment. All experimental procedures were approved by the animal management committee of China Medical University (IACUC approval number 2018-280).

### 2.7. Experimental Design

After 1 week of adaptive feeding, all mice were randomly divided into 7 groups (*n* = 6): (1) saline, (2) cisplatin (20 mg/kg, i.p.), (3) amifostine (AMF; 200 mg/kg, i.p.; dissolved in normal saline)+cisplatin (20 mg/kg), (4) ECC (250 mg/kg; dissolved in carboxymethyl cellulose (CMC))+cisplatin (20 mg/kg), (5) ECC (500 mg/kg)+cisplatin (20 mg/kg), (6) WCC (250 mg/kg; dissolved in CMC)+cisplatin (20 mg/kg), and (7) WCC (500 mg/kg)+cisplatin (20 mg/kg) groups. The mice in the treatment groups were orally administered ECC or WCC for 10 consecutive days. Normal mice were treated with normal saline. On the seventh day, animals in the cisplatin group and ECC- and WCC-treated groups were given a single dose of cisplatin (20 mg/kg, i.p.), 30 min after the administration of ECC or WCC, to induce kidney injury in mice. Three days after the injection of cisplatin, whole blood was collected and the mice were sacrificed. Serum was separated from blood by centrifugation at 4°C (2000 g, 15 min) and stored at -20°C. The kidneys were collected quickly for subsequent measurement. Treatment dose levels were selected based on the safety results of *C. cicadae* mycelia in chronic toxicity studies [20]. Clinical symptoms were observed twice daily during the study. Average measurements of body weight and food intake were conducted weekly during the study period.

### 2.8. Assessment of the Kidney/Body Mass Index

The body weight of the mice was measured before euthanasia. After euthanasia, the kidneys were surgically removed and weighed. The kidney/body mass index was calculated as follows: (kidney weight/body weight) × 100%.

### 2.9. Renal Function Tests

Serum markers of renal function were determined with BUN and CRE assay kits using a chemical analyzer (Roche Diagnostics, Cobas Mira Plus, Germany), according to the manufacturer's instructions.

### 2.10. Histopathological Analysis

The kidney tissue was immobilized in 10% formalin and embedded in paraffin. Sections with a thickness of 5 *μ*m were cut and stained with H&E, then photographed using light microscopy (Nikon Eclipse TS100, Japan). Renal injury was scored based on the percentage of epithelial necrosis in the cortical tubules: 0, normal kidney; 1, <25% damage; 2, 25–50% damage; 3, 50–75% damage; and 4, >75% damage [21].

### 2.11. Immunohistochemistry

Immunohistochemistry was carried out in 5 mm thick paraffin-embedded tissue sections. The primary antibody was rat polyclonal anti-F4/80 (1 : 50). Sections were used for immunohistochemical staining with the antibody. Color reaction was developed with diaminobenidine.

### 2.12. Lipid Peroxidation Assays

Malondialdehyde (MDA) levels, as a marker of lipid peroxidation in the kidneys, were measured using the thiobarbituric acid (TBA) reaction [22]. Briefly, 400 *μ*L of kidney extracts was mixed with 400 *μ*L of TBA reagent (0.4% TBA and 0.2% butylated hydroxytoluene). The mixture was placed in a 90°C water bath for 45 min, and an equal volume of n-butanol was added. The mixture was centrifuged, and the absorbance of the supernatant was recorded at 535 nm. A standard curve was obtained with a known amount of 1,1,3,3-tetraethoxypropane (TEP) using the same assay procedure. The levels of lipid peroxidation were expressed in terms of thiobarbituric acid reactive substances (TBARS) (nmol/mg protein).

### 2.13. Cytokine Assay

Serum TNF-*α*, IL-1*β*, and IL-6 were evaluated with an ELISA kit (BioLegend, San Diego, CA, USA), according to the manufacturer's instructions [23].

### 2.14. Glutathione Estimation

GSH content was measured with the GSH reductase-5,5′-dithiobis (2-nitrobenzoic acid) (DTNB) assay [22]. The tissues were homogenized with 10% trichloroacetic acid buffer and centrifuged at 1500 g and 4°C for 10 min. The reaction mixture contained 0.1 mL of supernatant, 2.0 mL of 0.3 M phosphate buffer (pH 8.4), 0.4 mL of double-distilled water, and 0.5 mL of DTNB. OD was used as a measure against a reagent blank after the addition of DTNB at 412 nm. GSH concentration was determined by comparing to a standard curve with a known amount of GSH.

### 2.15. Measurement of Intracellular ROS

Incorporation of DCFH-DA into cells leads to its conversion to 2,7-dichlorofluorescein (DCF) by oxidative processes. RAW264.7 cells were incubated with or without samples and then continuously stimulated with LPS (100 ng/mL) for 24 h. After washing thrice with phosphate-buffered saline, the cells were treated with serum-free medium containing 10 *μ*M H_2_DCFDA (Invitrogen, Carlsbad, CA, USA) for 30 min at 37°C in the dark and washed again with phosphate-buffered saline three times; then, the DCF fluorescence was detected by using a Synergy HT Microplate Reader (BioTek Instruments) with an excitation wavelength of 485 nm and emission wavelength of 535 nm [24].

### 2.16. Western Blot Analysis

For Western blot analysis, the tissue was homogenized and lysed in RIPA buffer with protease inhibitors, followed by centrifugation (12,000 × *g*, 20 min). The protein concentration was determined with a Bio-Rad protein assay kit (Bio-Rad, Hercules, CA). The proteins (50 *μ*g/lane) were then electrophoresed on 12% SDS polyacrylamide gel and transferred to a PVDF membrane. After blocking, the membranes were incubated with a primary antibody at 4°C. Appropriate horseradish peroxidase- (HRP-) conjugated secondary antibodies (Sigma, St. Louis, MO, USA) were applied, and the signals were detected using an ECL substrate (Amersham International plc., Buckinghamshire, UK). The Western blot analysis was performed using KODAK Molecular Imaging Software (Eastman Kodak Company, Rochester, NY, USA).

### 2.17. HPLC Analysis

Adenosine and N6-(2′-hydroxyethyl) adenosine (HEA) were determined by HPLC. WCC and ECC were redissolved in 20 volumes of solvent, and the supernatant was centrifuged by ultrasonic wave and filtered through a 0.45 *μ*m filter. HPLC (Hitachi L-5000 Series) with UV wavelength 254 nm was carried out using a reverse-phase separation column (Inertsil®, ODS-2, 4.6 × 250 mm, 5 *μ*m). This method involved the use of a binary gradient containing a mobile phase: (A) deionized water and (B) acetonitrile. The solvent gradient elution procedure was as follows: gradient 0–15 min 100% A, 15–40 min 100–80% A, 40–55 min 80–5% A, 55–75 min 50–0% A, 75–90 min 0% A, 90–91 min 0–100% A, and 91–100 min 100% A. The flow rate was 1.0 mL/min, and 10 *μ*L was injected, including the standards and samples.

### 2.18. Statistical Analysis

Values are expressed as mean ± SD for each group. Statistical analysis was performed by analysis of variance with a multiple comparison test (one-way analysis of variance (ANOVA) or Student's *t*-test). A *p* value less than 0.05 was considered to be significant.

## 3. Results

### 3.1. *C. cicadae* Mycelium Extract Reduced Renal Dysfunction and Histopathological Changes in Cisplatin-Induced Mice

**Figure 1:**
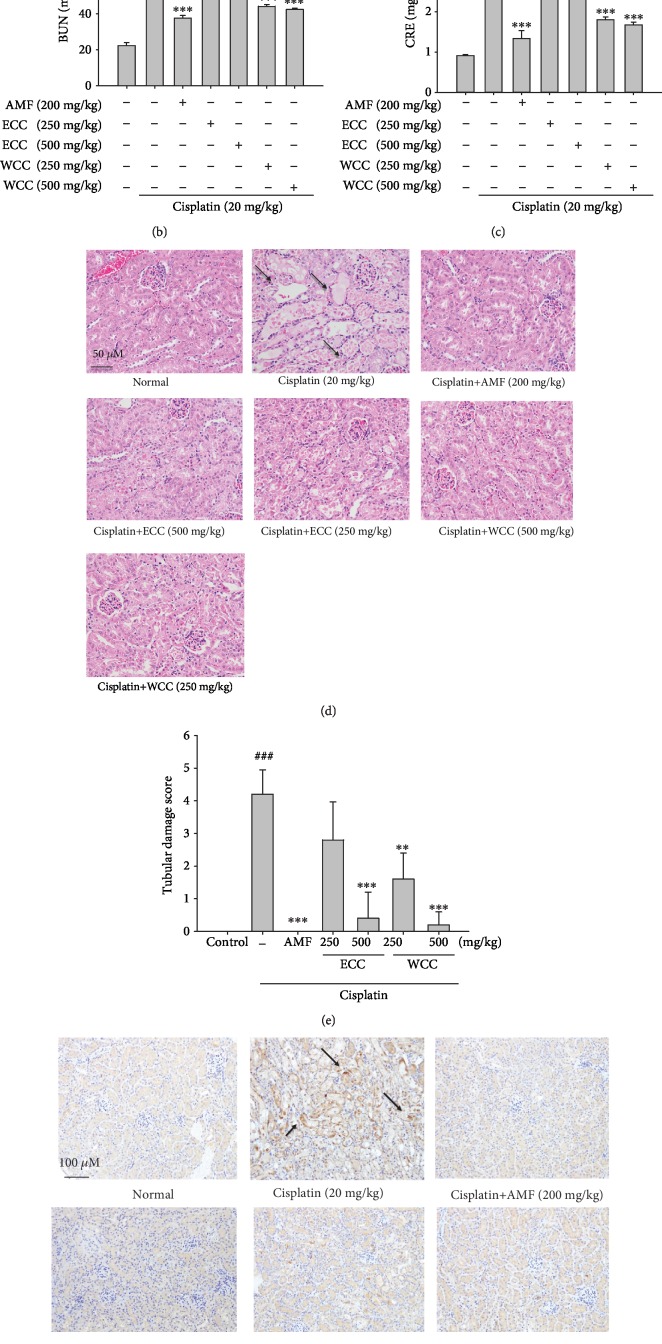
Protective effects of WCC and ECC on cisplatin-induced kidney damage in AKI mice. Mice were orally administered WCC and ECC daily for 10 days, they received cisplatin (20 mg/kg, i.p.) one hour after WCC and ECC administration on the seventh day, and the mice were euthanized on the eleventh day. The morphological changes in the kidneys (a). Blood urea nitrogen (BUN) levels (b). Serum creatinine (CRE) levels (c). Kidneys stained with H&E (d) and the tubular injury scores (e). Macrophage infiltration in kidney tissues was detected by immunohistochemistry with the F4/80 antibody (f). After cisplatin challenge, kidneys in each group were prepared for histological evaluation. Representative histological section of the kidneys was stained by H&E staining (magnification (400x)) and F4/80 immunohistochemical staining (magnification (200x)). The data are presented as the means ± SEM (*n* = 5). ^###^*p* < 0.001 compared with the sample of the control group. ^∗∗^*p* < 0.01 and ^∗∗∗^*p* < 0.001 compared with the cisplatin group. Tubular cell necrosis is marked with arrows. The bar indicates 50 *μ*m.

The morphological changes in the kidneys are shown in Figure 1(a). CRE and BUN are hallmarks of kidney function. Figures 1(b) and 1(c) show that, compared to the control group, cisplatin injection at a dose of 20 mg/kg highly increased the serum CRE (from 0.91 ± 0.03 mg/dL to 3.33 ± 0.41 mg/dL) and BUN (from 22.4 ± 1.63 mg/dL to 90.72 ± 3.67 mg/dL) levels (*p* < 0.001), indicating the generation of nephrotoxicity in the cisplatin-treated mice. Pretreatment with the *C. cicadae* mycelium water extract (WCC) and ethanolic extract (ECC) at doses of 250 and 500 mg/kg exerted a significant renal protection effect in a dose-dependent manner, as demonstrated by the normalization of CRE and BUN (*p* < 0.001) compared to the cisplatin-stimulated group. Amifostine (AMF) was used as a positive control. AMF is an inorganic thiophosphate cytoprotective agent, which has been evaluated as a cytoprotective agent against the toxicities of alkylating drugs and cisplatin.

Further, we analyzed the histopathological changes to determine whether WCC and ECC affected renal failure in cisplatin-stimulated mice. The kidney tissue of the control group was completely normal and characterized by a transparent tubular and glomerular structure with clear and normal nuclei. Kidneys had severe kidney damage in the cisplatin-stimulated mice, inducing tubular epithelial damage, inflammatory cell infiltration, tubular cell swelling, formation of intratubular casts, and tubular dilatation. However, pretreatment with WCC and ECC significantly improved necrosis and inflammatory infiltrating cells in the kidney tissue (see Figures 1(d) and 1(e)). F4/80-positive macrophages were observed in the kidney tissues in the AKI group, while WCC, ECC, and AMF decreased the F4/80-positive macrophage infiltration (Figure 1(f)).

### 3.2. *C. cicadae* Mycelium Extract Protected Mice from Cisplatin-Treated Changes on Morphological Changes of Kidney Tissues and Kidney Index

Cisplatin induction resulted in significant weight loss and an increase in the relative kidney index. Cisplatin-treated mice had a significant (*p* < 0.001) decrease in the body weight and relative kidney weight, as compared with normal mice (Table 1). However, the mice treated with ECC and WCC had significant (*p* < 0.001) protection from such a reduction in the body weight and kidney index.

### 3.3. *C. cicadae* Mycelium Extract Alerted the Cisplatin-Induced Changes in Proinflammatory Cytokines and NO

**Figure 2:**
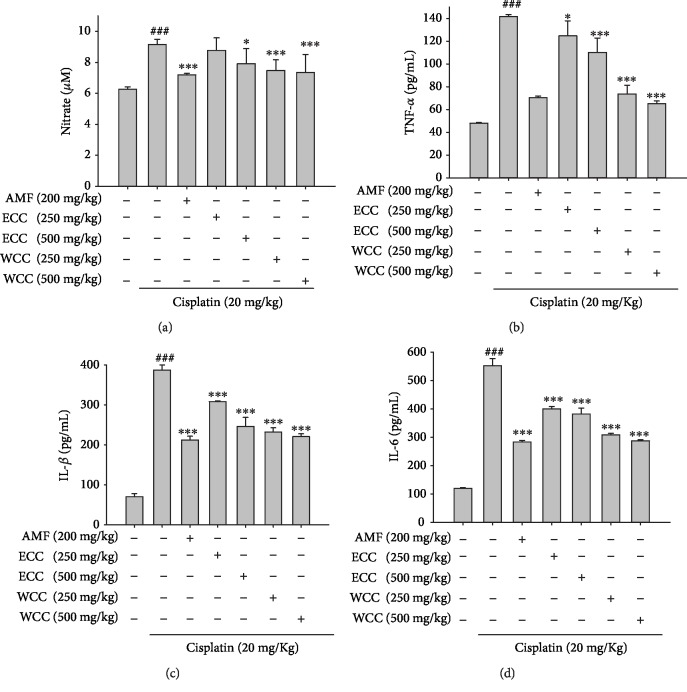
*C. cicadae* mycelium extract downregulated (a) NO, (b) TNF-*α*, (c) IL-1*β*, and (d) IL-6 in serum. Serum was collected. Nitrite concentration in the serum was measured by using the Griess reaction. Serum levels of TNF-*α*, IL-1*β*, and IL-6 were determined with commercial ELISA kits. Data are represented as mean ± SEM (*n* = 5). ^###^*p* < 0.001 compared with the sample of the control group. ^∗∗^*p* < 0.01 and ^∗∗∗^*p* < 0.001 compared with the cisplatin-only group.

Evaluation of levels of NO and proinflammatory cytokines TNF-*α*, IL-1*β*, and IL-6 in serum was performed by ELISA. Cisplatin-treated kidney injury mice had significantly increased NO, TNF-*α*, IL-1*β*, and IL-6 levels in serum, compared to the control group (Figures 2(a)–2(d), respectively). ECC, WCC, and AMF treatment improved NO, TNF-*α*, IL-1*β*, and IL-6 production after cisplatin challenge.

### 3.4. Effect of WCC on Oxidative Stress in the Kidney of Cisplatin-Treated Mice

**Figure 3:**
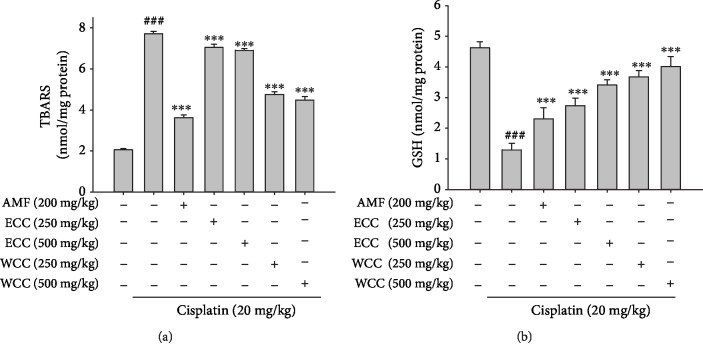
The effect of WCC on the oxidative stress in the kidney of cisplatin-treated mice. MDA levels (a). GSH levels (b). Kidney tissue homogenates were evaluated by the MDA and GSH assay. GSH was determined and expressed as *μ*mol/g kidney tissues. Data are represented as mean ± SEM (*n* = 5). ^###^*p* < 0.001 compared with the sample of the control group. ^∗∗^*p* < 0.01 and ^∗∗∗^*p* < 0.001 compared with the cisplatin-only group.

Oxidative stress is involved as a mechanism of cisplatin-induced kidney injury. As indicated in Figures 3(a) and 3(b), compared with the control group, cisplatin treatment caused a significant decrease in glutathione (GSH) levels and an increase in MDA levels. However, administration with 250 and 500 mg/kg of WCC reduced MDA levels and recovered antioxidant ability, as illustrated by the improvement of GSH content. These data suggest the renal protection activity of WCC against cisplatin-induced kidney injury. In addition, all of the above experiments showed that inhibition effects of WCC, as indicated by the serum markers BUN, CRE, NO, TNF-*α*, IL-1*β*, and IL-6, as well as GSH and MDA levels in the kidney, were superior to those of ECC. Thus, further experiments were carried out to analyze WCC.

### 3.5. Inhibition of Cisplatin-Induced Renal Injury iNOS and COX-2 and Inhibition of TLR4, p-I*κ*B*α*, I*κ*B*α*, p-NF-*κ*B, and NF-*κ*B Protein Expression

**Figure 4:**
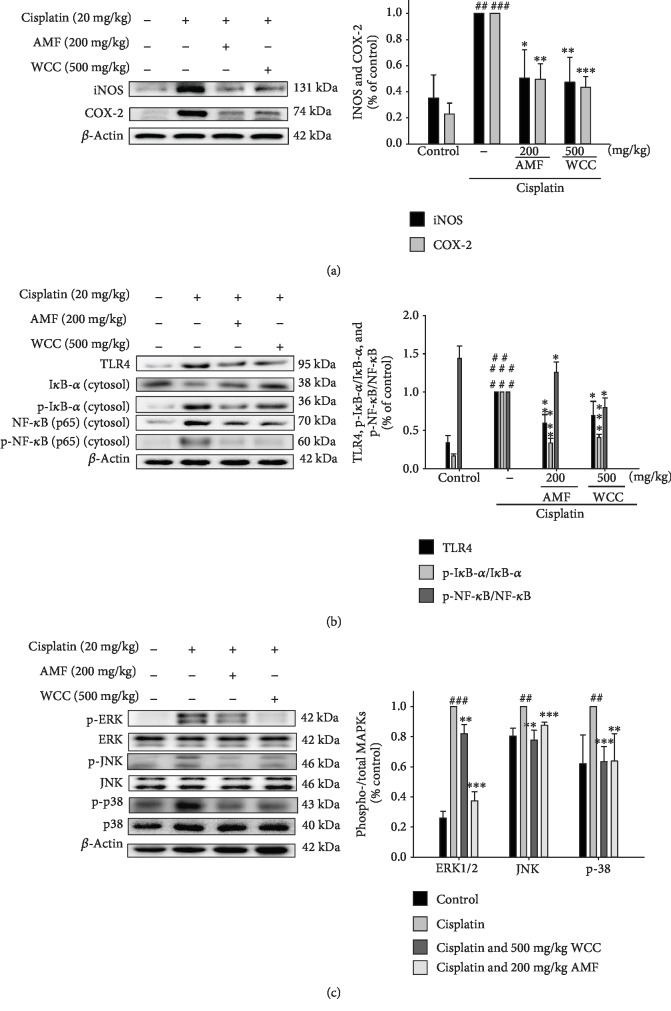
Effects of WCC on cisplatin-induced (a) iNOS, COX-2, (b) TLR4, I*κ*B*α*, p-I*κ*B*α*, NF-*κ*B, p-NF-*κ*B, and (c) MAPK phosphorylation signaling expression in kidneys. Protein levels of iNOS, COX-2, TLR4, I*κ*B*α*, NF-*κ*B, p-I*κ*B*α*, p-NF-*κ*B, and MAPK phosphorylation protein expression in kidney homogenates were evaluated by Western blot analysis after cisplatin challenge. Densitometric analysis of the relevant bands was performed. Data are represented as mean ± SEM (*n* = 3). ^###^*p* < 0.001 compared with the control group. ^∗∗^*p* < 0.01 and ^∗∗∗^*p* < 0.001 compared with the cisplatin-only group.

We examined whether pretreatment with *C. cicadae* mycelium extract WCC inhibited cisplatin-induced iNOS and COX-2 protein expression. The results revealed that pretreatment with WCC inhibited the protein expression of iNOS and COX-2 in the kidney tissues after the cisplatin challenge (Figure 4(a)).

Toll-like receptors (TLRs) are widely distributed on renal epithelial cells, with the function of regulating innate and adaptive immune responses. We examined the role of the TLR4 signaling pathway in cisplatin-induced renal injury. The results demonstrate that WCC treatment inhibited TLR4 expression in cisplatin-challenged mice (Figure 4(b)). Thus, WCC regulates the TLR4 signaling pathway after cisplatin challenge.

Signal pathways causing NF-*κ*B accumulation in the nucleus can be activated, by various stimuli, through the proinflammatory cytokines TNF-*α* and IL-1*β* [19]. Our study demonstrates that WCC treatment inhibited p-NF-*κ*B, NF-*κ*B, p-I*κ*B*α*, and I*κ*B*α* degradation in cisplatin-challenged mice (Figure 4(b)). Thus, WCC regulates the NF-*κ*B signaling pathway after cisplatin challenge.

### 3.6. Suppression of the MAPK Pathway

As shown in Figure 4(c), we found that the phosphorylation of MAPK (i.e., extracellular receptor kinases (ERK1/2), the c-Jun N-terminal kinases (JNK), and p38 mitogen-activated protein kinases (p38)) proteins occurred after cisplatin challenge in mice. However, the expression of phosphorylated ERK, JNK, and p38 decreased when pretreated with WCC and AMF. These results indicate that WCC inhibits the expression of MAPK proteins in cisplatin-treated mice.

### 3.7. Decreased Cisplatin-Treated Oxidative Stress and HO-1/Nrf2 Signaling Pathway

**Figure 5:**
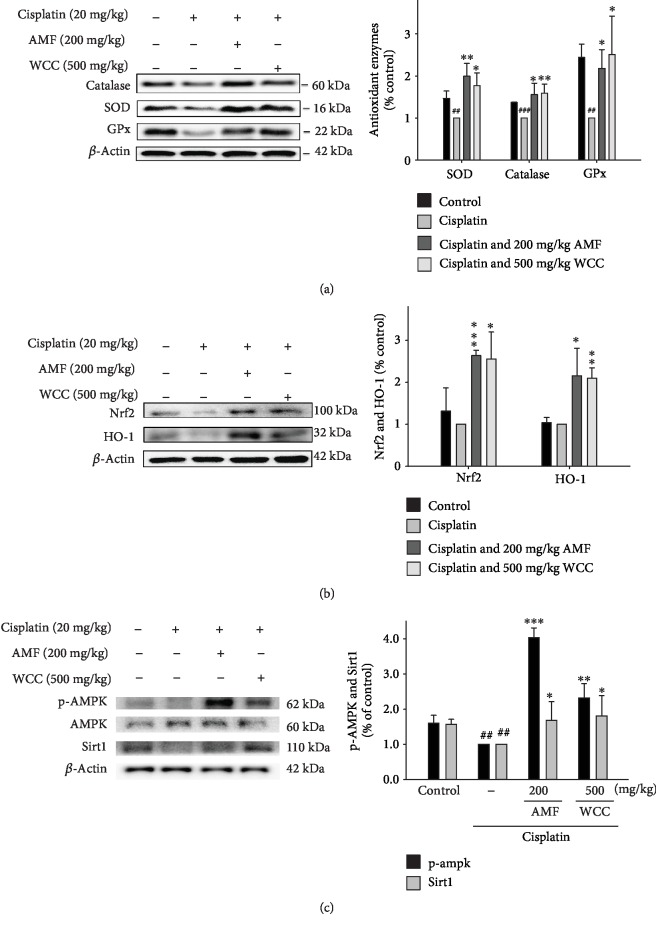
Effects of WCC on (a) cisplatin-induced antioxidative enzyme (catalase, SOD, and GPx), (b) HO-1, Nrf2, (c) AMPK, and Sirt1 protein expression and in the renal tissues. The protein levels of antioxidative enzyme, HO-1, Nrf2, AMPK, and Sirt1 protein expression in kidney homogenates were evaluated by Western blot analysis after cisplatin challenge. Densitometric analysis of the relevant bands was performed. Data are represented as mean ± SEM (*n* = 3). ^###^*p* < 0.001 compared with the control group. ^∗∗^*p* < 0.01 and ^∗∗∗^*p* < 0.001 compared with the cisplatin-only group.

Oxidative stress elevates the levels of reactive oxygen species, which can cause tissue injury. Superoxide dismutase (SOD) is a class of related enzymes that catalyze the breakdown of superoxide anions, and SOD activity has been shown to be reduced in cisplatin-treated mice [25]. Administration of cisplatin reduced the activity of catalase, SOD, and glutathione peroxidase (GPx) (Figure 5(a)), as well as reducing the antioxidative relative protein expressions of the HO-1 and Nrf2 proteins, compared with the control group (Figure 5(b)). However, WCC increased the antioxidant enzyme activities and the antioxidative relative protein expressions compared to cisplatin only. These results demonstrate that WCC improved the expression of antioxidative enzyme-related proteins after cisplatin challenge.

### 3.8. Decreased Cisplatin-Treated Sirt-1/AMPK Signaling Pathway

Phosphorylation of AMPK has been shown to increase SIRT1 expression under oxidative stress conditions to regulate energy homeostasis and metabolic stress [12]. In our study, decreased p-AMPK and Sirt1 levels were observed in cisplatin-treated mice. WCC upregulated p-AMPK and Sirt1 at the protein level in the kidneys of cisplatin-treated mice. AMF administration also restored the reduction of p-AMPK and SIRT1 levels induced by cisplatin exposure (Figure 5(c)). These results demonstrate that WCC improved the expression of p-AMPK and Sirt1 proteins after cisplatin challenge.

### 3.9. Decreased Cisplatin-Treated Apoptosis, Autophagy, and Anion Transport Protein Signaling Pathway

**Figure 6:**
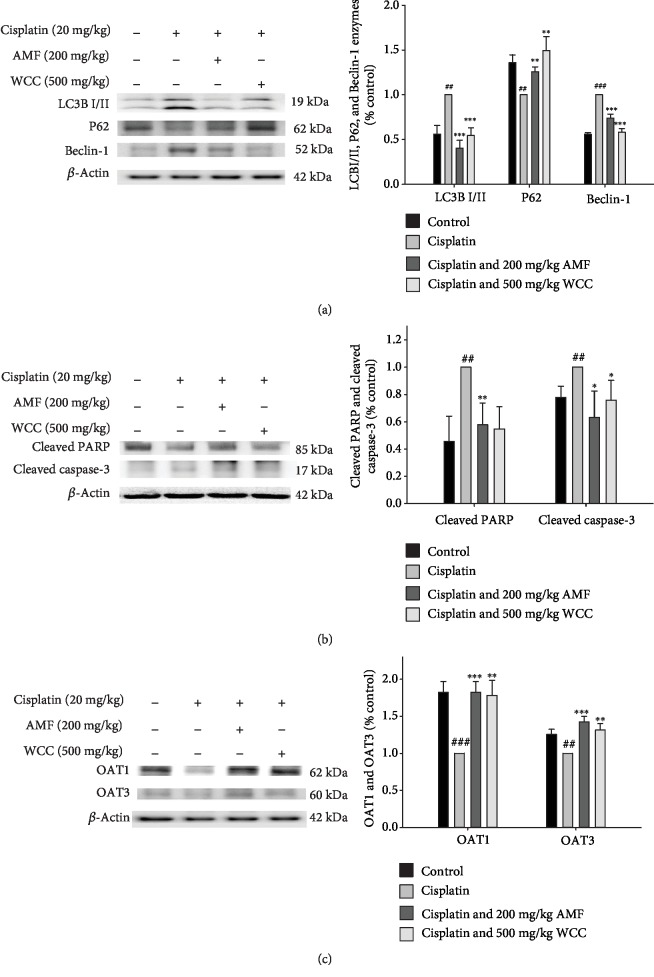
Effects of WCC on (a) cisplatin-induced apoptosis relative enzyme (cleaved caspase-3 and PARP), (b) autophagy relative enzyme (LC3B, p62, and Beclin-1), and (c) renal organic anion transporter (OAT1 and OAT3) protein expression and in the renal tissues. The protein levels of caspase-3 and PARP, LC3B, p62, Beclin-1, OAT1, and OAT3 protein expression in kidney homogenates were evaluated by Western blot analysis after cisplatin challenge. Densitometric analysis of the relevant bands was performed. Data are represented as mean ± SEM (*n* = 3). ^###^*p* < 0.001 compared with the control group. ^∗∗^*p* < 0.01 and ^∗∗∗^*p* < 0.001 compared with the cisplatin-only group.

Expression of the autophagy-associated proteins LC3B, p62, and Beclin-1 is associated with autophagy. We found that the levels of LC3B and Beclin-1 were significantly increased and p62 was decreased in cisplatin-treated mice, as compared to the control group, indicating a possible inhibition of autophagy. However, this effect was reversed by administration of 500 mg/kg WCC and in AMF-treated mice (Figure 6(a)). These results suggest that WCC could induce autophagy, thereby protecting the mice from cisplatin-mediated kidney injury.

PARP overactivation results from DNA damage and leads to cell apoptosis and necrosis, thereby participating in the development of disease. As shown in Figure 6(a), cisplatin injection caused an obvious increase in the cleaved PARP 1 and cleaved caspase-3 proteins. However, the levels of cleaved caspase-3 and PARP protein were effectively inhibited after pretreatment with WCC or with AMF administration (Figure 6(b)).

Drug transporters are important determinants of the clearance pathway from the systemic circulation and, consequently, play an important role in the pharmacological response. We found that WCC increased the expression levels of organic anion transporters 1 (OAT1) and OAT3 in the cisplatin-mediated kidney, compared with the control group (Figure 6(c)). The results suggest that WCC had an effect on the upregulation of OAT1 and OAT3.

### 3.10. *C. cicadae* Mycelium Extract Was Analyzed by HPLC

**Figure 7:**
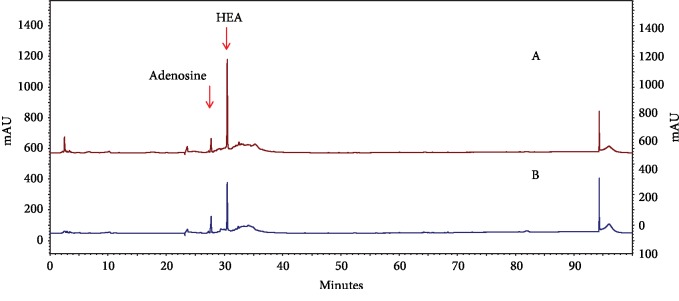
HPLC chromatogram of the *C. cicadae* mycelium water extract (WCC) (A) and ethanolic extract (ECC) (B).

Adenosine and HEA are the main pharmacologically active components of *C. cicadae* [17]. Therefore, their content in the mycelium extract was analyzed by HPLC. The amount of adenosine in the WCC extract (0.21 ± 0.01 mg/g) was lower than that in the ECC extract (0.24 ± 0.01 mg/g); further, the level of HEA in WCC extracts (1.65 ± 0.02 mg/g) was higher than that in the ECC extract (1.21 ± 0.03 mg/g) (Figure 7).

### 3.11. Effect of WCC, ECC, Adenosine, and HEA on LPS-Stimulated NO and ROS Production in Mouse Macrophage Cells

**Figure 8:**
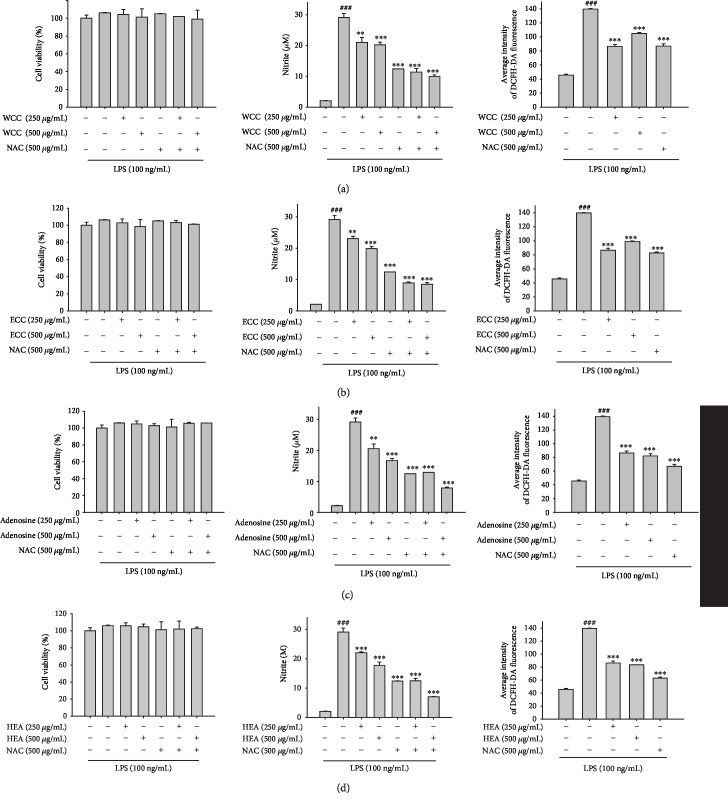
Effect of (a) WCC, (b) ECC, (c) adenosine, and (d) HEA on lipopolysaccharide- (LPS-) induced cell viability and NO and ROS production. Cells were incubated for 24 h with 100 ng/mL of LPS in the absence or presence of samples. Samples were added 1 h before incubation with LPS. The cell viability assay was performed using the MTT assay. Nitrite concentration in the medium was determined using the Griess reagent. Serum-free medium containing H_2_DCFDA 10 mM was added to cells, followed by 30 min incubation (37°C). 2,7-Dichlorofluorescein fluorescence was evaluated using a fluorescence ELISA reader. Densitometric analysis of the relevant bands was performed. Data are represented as mean ± SEM (*n* = 3). ^###^*p* < 0.001 compared with the control group. ^∗∗^*p* < 0.01 and ^∗∗∗^*p* < 0.001 compared with the cisplatin-only group.

The NO inhibitory activity of WCC, ECC, adenosine, and HEA was determined using LPS-activated macrophages to determine NO free radicals by the Griess reaction. WCC, ECC, adenosine, and HEA were found to reduce the NO production of activated macrophages in LPS-stimulated RAW264.7 cell lines, as shown in Figures 8(a)–8(d), respectively. This suggests that WCC, ECC, adenosine, and HEA may be better potential NO-related inflammatory pathway inhibitors. In addition, N-acetyl-L-cysteine (NAC) suppressed the generation of ROS in LPS-induced RAW264.7 cells. RAW264.7 cells were preincubated with NAC with or without WCC, ECC, adenosine, and HEA and then stimulated with LPS. We found that NAC with or without WCC, ECC, adenosine, and HEA markedly reduced the LPS-induced NO expression in RAW264.7 macrophages (Figures 8(a)–8(d)).

Increased ROS generation was observed in RAW264.7 cells stimulated with LPS, whereas the inhibitory effect was significantly blocked by WCC, ECC, adenosine, and HEA (Figures 8(a)–8(d)). Thus, LPS-mediated ROS production was significantly inhibited by WCC, ECC, adenosine, and HEA.

## 4. Discussion

AKI is considered to be a major limiting lesion in the clinical application of cisplatin chemotherapy, leading to an increase in chronic kidney disease and mortality. In general, the development of new prevention strategies is very important and urgent, along with described mechanisms of action to prevent or reduce cisplatin-induced AKI [26]. Although little is known about the specific mechanisms of cisplatin-treated kidney damage, a growing body of evidence has suggested that oxidative stress and inflammation play a very important role in cisplatin-treated AKI [27]. In this study, we induced AKI in a mouse model using intratracheal administration of cisplatin to confirm the protective effects on oxidative stress and inflammation by the *C. cicadae* mycelium extract. However, the protective effects of the *C. cicadae* mycelium extract on cisplatin-induced nephrotoxicity remain unclear. The objective of this study was to investigate the protective effects of the *C. cicadae* mycelium extract on cisplatin-induced renal injury *in vivo*, where AMF was used as a positive control group. The mechanism of action of AMF is by phosphorylation of alkaline phosphatase into its active metabolite, which is free sulfhydryl [28]; free sulfhydryl-binding free radicals mediate the protective effects. It has been reported that AMF can selectively protect normal cells from chemotherapy and radiotherapy and may improve the treatment by reducing dose-limiting toxic effects in preclinical studies [29]. Moreover, AMF has been shown not to change the antitumor effects of chemotherapy or radiotherapy [29].

Three days after the single administration of cisplatin in mice, serum BUN and CRE increased, as compared with the normal group [6]. The cisplatin group was characterized by renal insufficiency, which was ameliorated by WCC and ECC pretreatment. These data showed that WCC and ECC could act as potential protectors against cisplatin-induced AKI. Furthermore, treatment with WCC and ECC for 10 days lowered CRE and BUN levels in cisplatin-exposed mice, indicating that WCC and ECC improved renal function. In addition, the study found that the renal protection effect of WCC was better than that of ECC, as the kidney index, CRE, and BUN in the WCC group were significantly lower than those in the ECC group after exposure to cisplatin.

Administration of cisplatin has been shown to lead to significant renal impairment and histological changes, such as cast formation, brush border membrane loss, and tubular dilatation [30]. In the present study, we observed tubular dilatation and severe tubular necrosis in the cisplatin control group. Furthermore, the protective effects of WCC and ECC were demonstrated by little histological change in the treatment groups. Administration of cisplatin failed to induce any histological changes in the treatment group, due to the protection provided by the WCC and ECC. There were few dilated tubules and tubular necrosis in the treatment group, confirming the protective effects of WCC and ECC on cisplatin-induced nephrotoxicity.

The pathogenesis of AKI induced by cisplatin is an inflammatory response [5]. It has been shown that inflammatory cells, such as macrophages of the immune system, can infiltrate renal tissues to cause damage to kidney tissues during cisplatin-induced AKI [31]. Although cisplatin can directly lead to drug accumulation and cytotoxicity in the proximal tubules, cytokines have been found to exacerbate kidney damage [6]. In particular, TNF-*α*, IL-1*β*, and IL-6 play a key role in cisplatin-induced kidney injury [32]. Therefore, we studied the changes in cytokine levels in the kidney after cisplatin injury. We found that, after cisplatin administration, TNF-*α*, IL-1*β*, and IL-6 levels in the kidney were significantly increased. In contrast, WCC and ECC inhibited the expression of TNF-*α*, IL-1*β*, and IL-6 in the kidney after cisplatin administration. Inhibition of the production of these cytokines may be another important mechanism by which WCC and ECC improve cisplatin-induced AKI.

The NF-*κ*B signaling pathway plays an important role in inflammatory responses induced by oxidative stress, cell proliferation, and differentiation. NF-*κ*B is a protein complex which regulates cellular signaling in a variety of conditions [33]. In the cytoplasm, NF-*κ*B is expressed in an inactive form and binds to the protein I*κ*B. Under certain conditions, NF-*κ*B is activated and nuclear genes, such as COX-2 and iNOS, are transcribed [34]. Regulation of iNOS and other inducible genes (such as COX-2) has been considered to be an important mechanism in the inflammatory response [35]. It was found that the expression levels of COX-2, iNOS, p-I*κ*B*α*, and NF-*κ*B proteins increased sharply in mice exposed to cisplatin, while WCC treatment inhibited such an increase. Current data indicate that WCC can protect against cisplatin-induced nephrotoxicity by inhibiting the inflammatory pathway.

Toll-like receptors (TLRs) are a family of receptors whose function is to promote the release of cytokines, such as TNF-*α* and IL-1*β* [36]. After stimulation, TLRs can induce inflammatory cytokine expression by MyD88-dependent and MyD88-independent signaling pathways. The most widely characterized TLR, TLR4, is a receptor for Gram-negative bacterial endotoxins [37]. TLR4 can also be activated by endogenous molecules released during tissue damage. In addition, a variety of cells including circulating and resident immune cells and renal parenchymal cells expressed TLR4. Activation of TLR4 in any cells could lead to inflammation reaction and subsequent renal injury [38]. In this study, our results showed that WCC could inhibit cisplatin-induced TLR4 expression. It is well known that NF-*κ*B plays a critical role in the regulation of inflammatory cytokine production. Studies showed that NF-*κ*B activation was involved in cisplatin-induced nephrotoxicity both in patients and in animal models [39]. Furthermore, inhibition of NF-*κ*B activation could protect mice from cisplatin-induced nephrotoxicity. Therefore, TLR4 may be activated by cytokine production during cisplatin nephrotoxicity and renal dysfunction.

The MAPK pathway is a key factor in regulating cisplatin-induced kidney damage and inflammation. Based on previous evidence, various biological responses have been associated with the activation of MAPKs, which can activate the MAPK pathway by producing oxidative stress and TNF-*α* in the kidney tissues [40]. In this study, we showed that there was an increased amount of the phosphorylated MAPK proteins in the kidneys of cisplatin-treated mice. Pretreatment with WCC blocked the activation of MAPKs, thereby providing a potential mechanism for the beneficial effects of WCC. WCC also suppressed the production of TNF-*α*, IL-1*β*, and IL-6 through NF-*κ*B activation, as it controls proinflammatory cytokine expression in the cisplatin-induced model. In this study, we discovered that WCC significantly inhibited I*κ*B*α* degradation and the phosphorylation of NF-*κ*B and MAPK in cisplatin-challenged mice.

Oxidative stress is one of the key factors in the pathogenesis of cisplatin-treated renal injury [40]. Cells express a number of antioxidant enzymes (e.g., SOD, catalase, and GPx) to prevent the cell or tissue damage caused by the expression of antioxidant enzymes, in an attempt to decrease oxidative stress. The function of SOD is to catalyze the distribution of superoxide anions to hydrogen peroxide and oxygen, whereas catalase and GPx catalyze hydrogen peroxide to form oxygen and water [41]. In this study, WCC increased the antioxidant protein activity in the AKI model. In addition, studies have shown that Keap1 is a key sensor for oxidative stress. Under a large amount of oxidative stress, Keap1 induces the release of Nrf2, which activates Nrf2 and its downstream-regulated genes in the nucleus. Nrf2 is correlated with the induction of HO-1, GPx, glutathione-S-transferase, and Trx-1, allowing for the scavenging of free radicals caused by oxidative damage in cells [42]. Nrf2 functions to maintain cellular homeostasis through its ability to regulate antioxidant proteins, detoxification enzymes, and other stress response proteins [34]. Our results indicate that the protective effects of WCC regulated the Nrf2/HO-1 signaling pathway in cisplatin-challenged mice. Nrf2 is a key molecule involved in targeting specific proteins in the NF-*κ*B pathway, which may be associated with inflammatory regulation. Cisplatin-induced kidney damage produces oxidative stress caused by large amounts of ROS and GSH depletion, decreasing the levels of antioxidant enzymes catalase, SOD, and GPx and leading to higher levels of MDA, the final product of lipid hydroperoxides. A similar phenomenon was observed in our study; however, WCC pretreatment effectively reversed these oxidative stress changes to relatively normal levels.

The AMPK/Sirt1 pathway has been shown to be involved in the regulation of proinflammatory cytokine release [43]. AMPK is an energy-sensitive serine/threonine protein kinase widely found in eukaryotes, which is essential for mitochondrial biogenesis to regulate energy metabolism and respond to energy deprivation. It has also been found that Sirt1 regulates intracellular metabolism and attenuates ROS-induced apoptosis, resulting in longevity and acute stress resistance. Therefore, the AMPK/Sirt1 pathway has become an interesting target, due to its large involvement in catabolism, mitochondrial activation, and increased cell viability [44]. It is well known that the effects of resveratrol and n-3 polyunsaturated fatty acid (n-3 PUFA) are mediated by both Sirt1 and AMPK [45]. However, the direct relationships between kidney-specific Sirt1, AMPK, and renal tissue survival *in vivo* have not yet been elucidated. In this study, a Western blot experiment showed that cisplatin-induced AKI reduced the expression of Sirt1 and AMPK, which may be attenuated after treatment with WCC.

One of the goals of this study was to discuss the possible mechanisms by which cisplatin promotes autophagy in cisplatin-induced AKI, where autophagy is an important protective mechanism for cell survival. It can protect the proximal tubules of the kidney from AKI by eliminating the mitochondria that produce ROS during CP treatment. Autophagy involves many important molecules, such as Beclin-1 (autophagosome nucleation) [13], LC3B (autophagosome membrane expansion and fusion), and p62 (cargo receptors involved in selective autophagy) [14]. The effects of WCC on macrophage autophagy were thus studied. WCC induced primary macrophage occurrence in experimental mice by increasing LC3B and Beclin-1 expression, as well as decreasing p62 expression.

Membrane transporters are important in the kidney for the absorption and removal of chemicals and metabolic byproducts [46]. It has been reported that OATs, mediated by organics secreted by the kidney, reduce the incidence of renal failure [47]. Among them, the membrane transporters OAT1 and OAT3 are expressed in the basolateral membrane of renal proximal tubule cells. In addition, cisplatin-induced nephrotoxicity has been shown to reduce OAT1 and OAT3 transporters [15]. In this study, the combination of WCC and cisplatin significantly increased the expression of OAT1 and OAT3 in mice.

## 5. Conclusions

**Figure 9:**
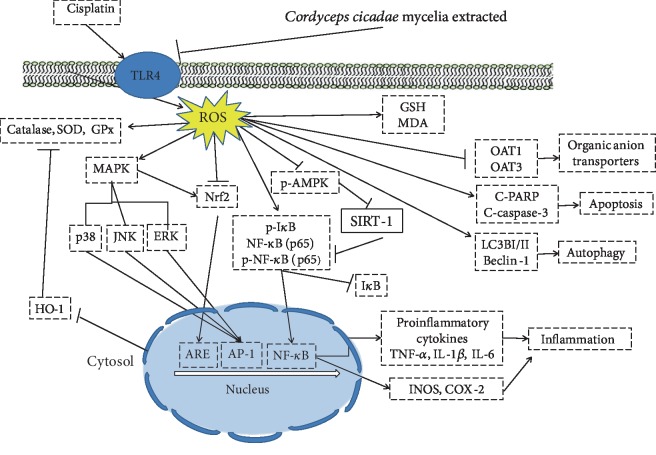
The schemes of the mechanism for the protective effect of WCC in cisplatin-induced kidney injury.

Our study revealed, for the first time, that the *C. cicadae* mycelium extract regulates inflammatory responses in a cisplatin-induced AKI animal model by inhibiting renal pathologic changes, inflammatory cell infiltration, and the release of a variety of proinflammatory cytokines (i.e., TNF-*α*, IL-1*β*, and IL-6). The research data suggest that the *C. cicadae* mycelium extract possesses potent anti-inflammatory properties, which were mediated by inhibiting the TLR4/NF-*κ*B/MAPK, HO-1/Nrf2 and AMPK/Sirt1 signaling pathways (Figure 9). In addition, the *C. cicadae* mycelium extract-mediated alleviation of cisplatin-induced nephrotoxicity was (in part) due to regulation of autophagy, inhibition of apoptosis, and increasing OAT expressions in the kidney tissues. Therefore, the *C. cicadae* mycelium extract exerts anti-inflammatory effects *in vivo* and shows potential as a promising agent for clinical applications in the near future.

## Figures and Tables

**Table 1:** Effects of WCC and ECC on body weight change and kidney index in cisplatin-induced acute kidney injury. The data are presented as the means ± SEM (*n* = 5). ^###^*p* < 0.001 compared with the sample of the control group. ^∗∗∗^*p* < 0.001 compared with the cisplatin group.

Groups	Dosage (mg/kg)	Initial body (g)	Final body (g)	Kidney index (mg/g)
Normal	—	29.17 ± 0.35	31.8 ± 0.47	1.34 ± 0.03
Cisplatin	—	29.28 ± 0.83	23.05 ± 0.36	2.5 ± 0.08^###^
AMF	200	30.37 ± 0.39	28.4 ± 0.66	1.6±0.02^∗∗∗^
ECC	500	29.65 ± 0.52	27.62 ± 0.32	1.78±0.02^∗∗∗^
ECC	250	30.27 ± 0.4	27.98 ± 0.47	1.8±0.06^∗∗∗^
WCC	500	30.35 ± 0.48	28.62 ± 0.43	1.66±0.05^∗∗∗^
WCC	250	30.33 ± 0.54	26.88 ± 0.52	1.91±0.06^∗∗∗^

## Data Availability

The data used to support the findings of this study are available from the corresponding author upon request.
